# Barley’s Second Spring as a Model Organism for Chloroplast Research

**DOI:** 10.3390/plants9070803

**Published:** 2020-06-27

**Authors:** Lisa Rotasperti, Francesca Sansoni, Chiara Mizzotti, Luca Tadini, Paolo Pesaresi

**Affiliations:** Dipartimento di Bioscienze, Università Degli Studi di Milano, 20133 Milano, Italy; lisa.rotasperti@unimi.it (L.R.); francesca.sansoni1@studenti.unimi.it (F.S.); chiara.mizzotti@unimi.it (C.M.); luca.tadini@unimi.it (L.T.)

**Keywords:** barley, genome, functional genomics, chloroplast biogenesis, photosynthesis improvement

## Abstract

Barley (*Hordeum vulgare*) has been widely used as a model crop for studying molecular and physiological processes such as chloroplast development and photosynthesis. During the second half of the 20th century, mutants such as *albostrians* led to the discovery of the nuclear-encoded, plastid-localized RNA polymerase and the retrograde (chloroplast-to-nucleus) signalling communication pathway, while *chlorina-f2* and *xantha* mutants helped to shed light on the chlorophyll biosynthetic pathway, on the light-harvesting proteins and on the organization of the photosynthetic apparatus. However, during the last 30 years, a large fraction of chloroplast research has switched to the more “user-friendly” model species *Arabidopsis thaliana*, the first plant species whose genome was sequenced and published at the end of 2000. Despite its many advantages, Arabidopsis has some important limitations compared to barley, including the lack of a real canopy and the absence of the proplastid-to-chloroplast developmental gradient across the leaf blade. These features, together with the availability of large collections of natural genetic diversity and mutant populations for barley, a complete genome assembly and protocols for genetic transformation and gene editing, have relaunched barley as an ideal model species for chloroplast research. In this review, we provide an update on the genomics tools now available for barley, and review the biotechnological strategies reported to increase photosynthesis efficiency in model species, which deserve to be validated in barley.

## 1. Barley, the Crop and the Model Species

Barley (*Hordeum vulgare*) is a self-pollinating monocotyledonous plant species that belongs to the *Poaceae*, a grass family that includes several major crops exploited in modern agriculture. Its domestication dates back to 10,000 BC, took place in the Fertile Crescent and began with the wild species *Hordeum vulgare* ssp. *spontaneum* [1]. Barley ranks fourth in terms of annual grain tonnage after maize, wheat and rice, with a worldwide production level (2018/2019) of 141 million tons. The primary role of cultivated barley (*Hordeum vulgare* ssp. *vulgare*) is as a source of animal feed (about 75% of the global production), with subsidiary uses in alcoholic and non-alcoholic beverages (20%), and in human nutrition (5%)—partly due to its high content of beta-glucan, a beneficial fibre that can reduce levels of cholesterol in the blood. During the 20th century, barley was widely exploited as a model species for crop studies. As a self-pollinating species with a diploid (2n) genome and a haploid complement of only seven chromosomes, barley proved to be an excellent model organism for both basic and applied research. Furthermore, due to the fact that wild barley (*Hordeum vulgare* ssp. *spontaneum*) can grow in a wide range of environments and climates, from the Arctic Circle to the equatorial highlands, barley is cultivated more widely than any other major crop. This resilience relies on a wealth of natural genetic diversity which enables the plant to adapt effectively to various environmental challenges such as cold temperatures, drought, alkalinity and salinity, and makes it a perfect model species for investigating crop adaptation to abiotic stresses [2].

### 1.1. A Brief History of Genome Manipulation in Barley

*Hordeum vulgare* was one of the very first crops used in cereal improvement programs based on different induced mutation strategies. In 1930, Stadler studied the mutagenic effects of different types of radiation on maize and barley, describing chlorophyll-deficient and virescent phenotypes in seedlings [3]. In 1938, Nilsson-Ehle and Gustafsson tested X-rays and UV light on the barley cultivar (cv.) ‘Gull’ and isolated several mutants, which were named *albina*, *xantha*, *alboviridis*, *viridis, tigrina, striata* and *maculata*, categorizing them by their carotenoid and chlorophyll contents and distribution within the leaf blade [4]. The characteristics of several mutated lines were recognized as being very valuable for potential use in agriculture, since they exhibited alterations in grain yield, straw stiffness, straw length and tillering capacity, as well as changes in spike firmness, kernel maturation and pigmentation [5]. Later on, two varieties of barley ‘Trebi’ and ‘Moister’ were exposed to the radiation generated by the first aerial atomic explosion at Bikini atoll in 1946 [6]. Meanwhile, Gustafsson and Mackey applied mustard gas to barley to observe the effect of chemical mutagenesis [7], whereas Ehrenberg and collaborators tested various mutagenic compounds on barley and evaluated their impact on chlorophyll accumulation [8]. After these pioneering experiments, a broad range of chemical and physical mutagens were tested systematically. During this phase, alkylating agents able to generate G/C to A/T transitions in DNA, such as EMS (Ethyl Methane Sulfonate), ENU (N-nitroso-N-ethylurea), MNU (N-nitroso-N-methylurea), DES (diEthyl Sulfate) and sodium azide (NaN_3_), were widely used for the mutagenesis of barley. The first chemically induced barley variety, ‘Luther’, was released in the US in 1966. ‘Luther’ was obtained by exposing the variety ‘Alpine’ to DES. In 1965, in Czechoslovakia, the variety ‘Diamant’ was obtained after gamma-ray irradiation. This new variety was ~ 15 cm shorter than the parental ‘Valticky’ and displayed an increase in grain yield amounting to about 12% [9]. At around the same time, in the UK, ‘Golden Promise’ was registered. This semi-dwarf cultivar originated from exposure of the salt-sensitive variety ‘Maythorpe’ to gamma rays [10]. The generation of ‘Golden Promise’ represented an important step towards the development of tissue culture and barley transformation techniques (see below).

### 1.2. Early Studies and Milestones in Understanding of Chloroplast Biogenesis and Physiology in Barley

Genetic studies of barley have not been restricted to breeding programs. The plant has also been used as a model species to dissect the molecular mechanisms that underlie plant development and physiology and, for a large part of the 20th century, it served as a major experimental system for the investigation of chloroplast biogenesis and photosynthesis. In particular, several studies during the 1970s characterized different aspects of plastid structure and development, such as plastid growth, replication and differentiation. Dark-grown barley seedlings were used to determine the protochlorophyll content and structure of the etioplasts. Exposure to different light conditions allowed chloroplast development to be characterised from both structural and biochemical points of view [11,12,13,14].

The organization of chloroplast membranes was analysed in chloroplast preparations solubilised with digitonin and fractionated by electrophoresis, proving the existence of distinct sets of membranes [15]. The functionality and structural organization of thylakoids were also studied in barley mutants altered in chlorophyll biosynthesis [16] and revealed the impact of such changes on thylakoid membrane organization. For instance, the *chlorina-f2* mutant, which is impaired in chlorophyll b accumulation, led to the discovery of light-harvesting chlorophyll-binding proteins [17,18,19,20]. *Chlorina-f2* was also used to assess the impact of protein-chlorophyll complexes on the ultrastructure of thylakoid membranes, shedding light on the organisation of the photosynthetic apparatus [17,21]. In addition, the *tigrina-d* mutant [22], originally suggested to be involved in the early steps of tetrapyrrole biosynthesis prior to ALA formation, was recently identified as the barley orthologue [23] of the *FLU* gene of *Arabidopsis thaliana*, a nuclear-encoded, plastid-localized protein that plays a key role in the negative-feedback control of chlorophyll biosynthesis, with an essential role during the dark-to-light switch [24]. Moreover, the barley *xantha* mutants helped to elucidate key steps in chlorophyll biosynthesis [25]: *xantha-l* was shown to code for a mutated form of Mg-protoporphyrin IX monomethyl ester cyclase, while *xantha-f*, *-g*, and *-h* carry genetic lesions at three distinct loci encoding the three Mg-chelatase subunits [26,27]. 

From a physiological point of view, Smith et al. [28] documented changes in chloroplast activity during de-etiolation of barley seedlings by measuring the Hill reaction in relation to chlorophyll accumulation. The correlation between plastid ultrastructure, chlorophyll synthesis and development of photosynthetic activity was also evaluated by measuring O_2_ evolution [29]. 

Besides the characterization of the photosynthetic apparatus, barley played an important part in the dissection of the chloroplast’s gene expression machinery. Indeed, evidence for a fully nuclear-encoded transcriptional activity in plastids, later named the Nuclear-Encoded RNA Polymerase (NEP; [30]), was first reported in barley, based on analysis of the *albostrians* mutant. In particular, the synthesis of RNA was reported in the white sectors of *albostrians* leaves, which harbor plastids that are devoid of ribosomes. These data provided initial evidence for the existence of a nuclear-encoded and plastid-localized RNA polymerase [31]. In addition, ribosome-free plastids of *albostrians* were helpful in distinguishing between the set of plastid genes preferentially transcribed by NEP, such as *rRNA*, *rpo* and *rps15*, and the set transcribed by the Plastid-Encoded RNA polymerase (PEP), which is enriched in photosynthesis-related genes such as *psbA*, *rbcL*, *atpI-H* [31,32]. Furthermore, the barley *albostrians* mutant was essential to the initial detection of communication between organellar and nuclear genomes. By analyzing *albostrians*, which is characterized by reduced amounts and/or activities of nucleus-encoded chloroplast proteins including the small subunit of ribulose-1,5-bisphosphate carboxylase⁄oxygenase (Rubisco), ferredoxin NADP+ reductase, and enzymes of the Calvin cycle, Börner provided the first evidence for plastid signals that control nuclear gene expression, leading to the discovery of chloroplast-to-nucleus retrograde communication [33,34,35]. 

## 2. *Arabidopsis thaliana* as the Model Plant of Modern Times

In the 1990s, crop models lost their dominant position in basic research on plants to *Arabidopsis thaliana*, which has now reigned supreme for three decades. Its small size, short life cycle, ability to produce thousands of seeds from a single plant and simple growth requirements were perfectly compatible with lab facilities and research workflows. Moreover, its small diploid nuclear genome (~135 Mb on 5 chromosomes) and the *Agrobacterium tumefaciens*-based transformation protocol made Arabidopsis ideal for use in basic research [36]. The Arabidopsis Genome Project was initiated in 1990, and led to the publication of the first sequenced plant genome in 2000 [37]. This, together with large collections of insertional mutants (SIGnAL, http://signal.salk.edu/cgi-bin/tdnaexpress), permitted the functional characterization of large numbers of genes and biological processes, thus laying the foundations for the modern age of plant biology. 

Although Arabidopsis has been considered the “golden” model species in plant science, it does have some limitations in terms of the extent to which lessons on development and physiology learnt from this model species can be extrapolated to monocots, including barley. This is particularly true for processes such as chloroplast biogenesis and photosynthesis. For instance, Arabidopsis does not produce an overhead canopy, and therefore cannot be employed in studies of plant architecture and optimization of photosynthesis under field conditions [38,39]. Moreover, the biogenesis of the multisubunit photosynthetic complexes, and indeed the chloroplast more generally, appear to differ significantly between monocotyledonous and dicotyledonous plants [40]. In monocots, the process of chloroplast development from the proplastid to functional chloroplasts can be observed as a gradient along the leaf blade, since leaves have a basal meristem and, as a consequence, the youngest cells carrying proplastids are found at the leaf base, while the leaf tip consists of the oldest cells with mature chloroplasts [29,41,42]. In contrast, in dicots like *Arabidopsis thaliana* chloroplast development varies between plant organs—i.e., between cotyledons and leaves—and with respect to the leaves, most of the events take place inside the shoot apex, which constitutes a major limitation for functional studies [43,44]. In light of these limitations, the widely accepted knowledge transfer route from Arabidopsis to crops is not always the most convenient and effective strategy, especially in the era of next-generation sequencing technologies and gene-editing approaches that make functional genomics studies feasible in species with complex genomes.

## 3. The Genomes of Barley 

### 3.1. The Nuclear Genome

**Figure 1:**
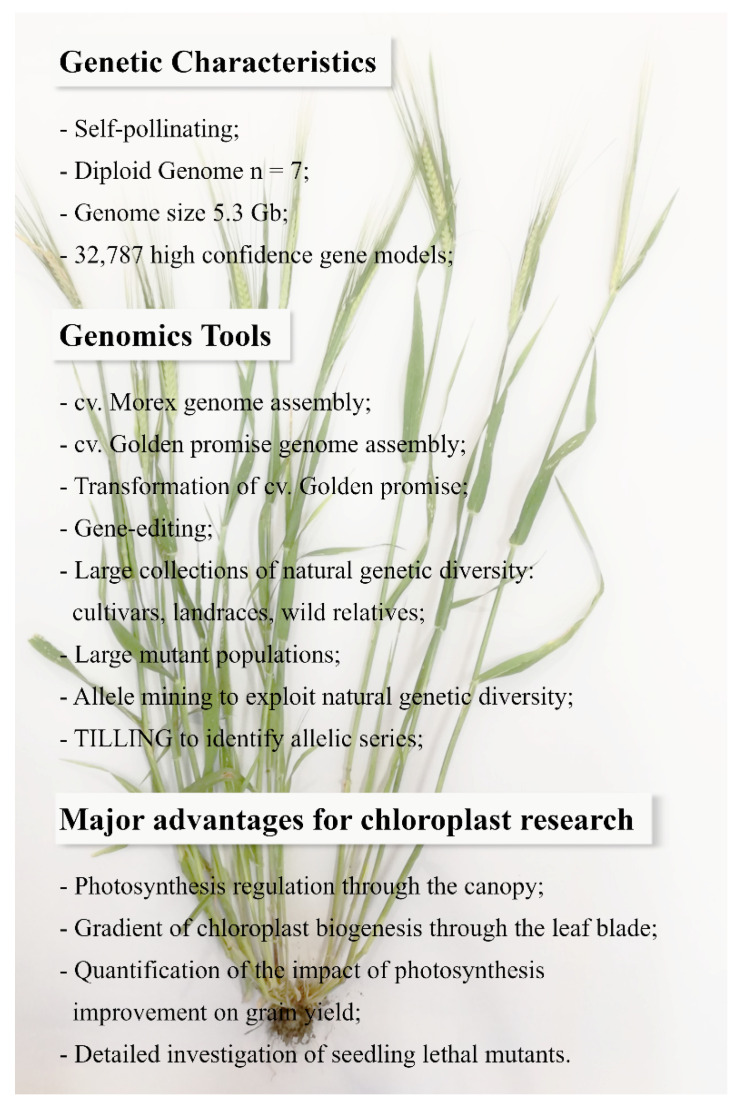
Overview of the genetic characteristics and genomics tools available for barley. These features, together with its canopy architecture and developmental properties, make barley an optimal model for chloroplast research.

For a long time, the absence of a reference genome has been the major obstacle to the exploitation of barley genomic resources in both research and breeding programs. The relatively large size of the barley genome (5.3 Gb), together with its high proportion of repetitive DNA (more than 80%), has severely compromised the assembly of the whole-genome shotgun sequence and the generation of a reference genome (Figure 1). However, in 2012 the International Barley Sequencing Consortium circumvented these problems by integrating several different strategies. This involved coupling a detailed physical map of the barley cv. ‘Morex’ (a US spring six-row malting barley) with high-density genetic maps, superimposing deep short-read whole-genome shotgun assemblies, and annotating the resulting linear genomic sequence with dense-coverage RNA-derived, i.e., full-length cDNA and RNA-seq, data. This strategy allowed approximately 4 Gb (80%) of the genome to be delineated, including more than 90% of the expressed genes, together with their physical distribution and patterns of expression [45]. This partially ordered sequence assembly has since been substantially improved by Mascher and collaborators [46] through the release of a map-based reference sequence of the barley cv. ‘Morex’ genome that included the first comprehensively ordered assembly of the pericentromeric regions. The final genome sequence covered 4.79 Gb (approximately 95% of the total genome size), of which 4.54 Gb were assigned to precise chromosomal locations. Mapping of transcriptome data and reference protein sequences from other plant species to the assembly identified 39,734 high- and 41,949 low-confidence genes, representing 98% of the Morex gene complement. Furthermore, homology-guided repeat annotation identified 3.7 Gb (80.8%) of the assembled sequence as derived from transposable elements, most of which were present as truncated and degenerated copies, with only 10% of mobile elements being intact and potentially active. A second improved version of the barley cv. ‘Morex’ reference genome has recently been released [47]. This is based on the use of TRITEX, an open-source computational workflow, whose output is available on the IPK Barley BLAST server (https://webblast.ipk-gatersleben.de/barley_ibsc/, see Table 1). The need for an improved assembly arose from large sequence gaps and local mis-assemblies present in the first reference sequence. A total of 32,787 high- and 30,871 low-confidence gene models were annotated in the second version of the barley genome. The smaller number of high-confidence gene models described in the second version of the genome (32,787 vs 39,734) is certainly due to the more precise annotation process, making the TRITEX-based assembly a greatly improved version of the reference genome. More recently, a reference genome assembly for the barley cv. Golden Promise has been reported [48]. The assembled genome of seven chromosomes comprising 4.13 Gb contains 95.2% of complete and single-copy genes and will prove particularly useful for functional genomics studies, given that Golden Promise is the most readily transformable barley genotype.

### 3.2. The Exomes

A broader knowledge of the genetic diversity of barley is an essential prerequisite for the development of new varieties with increased yields and greater environmental robustness. A comprehensive genotyping of germplasm based on exome sequencing currently offers the best route to this goal. Sequencing of the coding DNA alone dramatically decreases the complexity of the task, and reduces the computational effort and associated costs compared with whole-genome approaches. This makes it highly suitable for crops like barley, which contain very high proportions of transposon DNA [46]. The application of the exome approach to barley was initially reported in Mascher et al. [55], and was first applied to examine the crop’s adaptive responses in an analysis of 267 geo-referenced landraces and wild accessions [56]. This analysis combined exome capture and sequencing with field trials, bioclimatic data and various statistical approaches to investigate the genomic signatures that underlie barley’s adaptive responses to various environmental stresses. A total of 1,688,807 SNPs and 143,872 short InDels were identified in 59.5 Mb of genomic sequence. The study yielded a large pool of genetic variation to be exploited in future breeding programs, as many of the SNPs identified were rare, showing an overall allele frequency below 5% and being more highly represented in wild accessions. A similar strategy based on exome capture sequencing [57] explored the genetics of barley adaptation to multiple contrasting environments in 371 domesticated lines, comprising cultivars and landraces of both two- and six-rowed types. The identification of 435,431 SNPs uncovered significant genetic diversity—including a well-defined subset of spring-growth-habit barleys, made up of 111 cultivars and 63 landraces—as well as revealing strong differentiation at specific chromosome positions between two- and six-row barley lines, and high adaptation and heritability of phenotypes such as days to heading, plant height, 1000-grain weight and awn length.

## 4. Barley Genetic Resources: Natural and Induced Genetic Diversity

### 4.1. Natural Genetic Diversity

Crop improvement through crossing of high-performance cultivars has resulted in the loss of genetic diversity across cultivated genomes, a phenomenon known as the “domestication bottleneck” [58]. Therefore, landraces and wild accessions of barley are a precious pristine source of natural allelic variability that can be exploited in barley breeding programs, as has now been verified by exome sequencing assays (see above). Over the years, several research institutes around the world have collected barley accessions with the aim of preserving this genetic variability and making it accessible to breeders through the adoption of advanced methods that are better able to discover, dissect and deploy useful variations [46,59]. The major collections are maintained in institutions around the world, and the most representative are listed in Table 2. Among them, the ICARDA Collection hosts 222,704 barley accessions. Most of these are advanced materials, such as released cultivars and research lines, while 22% are geo-referenced wild barley relatives and landraces. The International Barley Core Collection (BCC) is a research collection that aims to represent the fullest possible range of the extant diversity of wild and cultivated barley. About 1300 accessions are currently available. Of these, some 300 varieties and landraces are held in the IPK Gatersleben Genebank. Of special relevance is the WHEALBI collection (http://www.whealbi.eu/), which comprises 511 accessions. This source of material represents a worldwide selection of barley’s genetic diversity, including landraces, cultivars and progenitors. In particular, the WHEALBI panel includes accessions originating from a wide range of locations covering key crop production regions in Europe, Africa, the Middle East and Asia. A subset of 371 domesticated lines chosen from the entire WHEALBI germplasm has been subjected to exome sequencing in order to correlate genomic and phenotypic data [57]. Various online platforms have been developed to facilitate searches of germplasm collections and provide detailed information on their origins and characteristics. Some of these are listed in Table 2. 

### 4.2. Induced Genetic Diversity: Random Chemical and Physical Mutagenesis

Besides natural genetic diversity, the availability of barley mutants is very important for understanding gene functions and their links with phenotypical traits (Figure 1). As described above (see Section 1.1), chemical and physical agents have been used to generate random mutagenized barley populations by several research groups. A few of these populations, derived from diverse genetic backgrounds, are listed in Table 3. Two large-scale EMS mutant populations from the cv. Optic, for instance, have been developed [60] and comprise approximately 20,000 M_2_ plants. TILLMore, a sodium azide-mutagenized population of cv. Morex, has also been created [61] and consists of 4906 M_3_ families. More recently, the *Hor*TILLUS (*Hordeum*—TILLING—University of Silesia) population, derived from the spring barley cultivar Sebastian following treatment of seeds with two chemical mutagens (NaN_3_ and MNU) and consisting of more than 9600 M_2_ plants, was reported [62]. However, one limitation of the available resources is that the parental cultivars used for mutant population development are all recalcitrant to genetic transformation. Consequently, gene-specific complementation assays, which are essential for phenotype-to-genotype association, are generally not possible. To mitigate this drawback, a heavily mutagenized EMS population of cv. Golden Promise (the reference variety used across the barley research community for genetic transformation and functional genomics) has been developed [63]. This population permits direct complementation of candidate mutations, opening up new possibilities for efficient functional genomics studies. 

During the last 15 years, mutagenized populations have emerged as a key resource for gene discovery [67]. Using forward genetics approaches, many genes, especially those that confer morphological or developmental phenotypes, have now been isolated [68,69,70,71]. In addition, Targeting Induced Local Lesions in Genomes (TILLING; [72]) has become particularly powerful for gene validation studies, and for exploring the roles of genes for which no obvious visual phenotype can be predicted, i.e., the reverse genetics approach [73]. Moreover, the TILLING approach produces allelic series, which are important for genes whose knock-out would be lethal, but also in cases where proteins/enzymes with novel properties are needed. Identification of the DNA sequence changes responsible for mutant phenotypes has been performed, so far, through heteroduplex analysis [74]. This involves amplification of the gene of interest from a DNA pool, enzymatic cleavage of heteroduplexes formed by allelic mismatches, and detection of the cleaved strands in polyacrylamide gels, followed by sequencing for confirmation of the variation. However, with the advent of Next Generation Sequencing (NGS) technologies and the availability of reference genomes, the use of exome capture sequencing and/or pooled amplicon sequencing of multiple target genes appears to be more effective in the case of barley [63,75]. Moreover, it is worth mentioning that these mutagenized barley plants are not considered as Genetically Modified (GM), and can be used in field trials to evaluate their performance, even in countries in which the cultivation of GM organisms is forbidden.

### 4.3. Induced Genetic Diversity: Genome Transformation and Insertional Mutagenesis 

The need for complete sequencing of the barley genome, the range of genetic diversity and the numerous mutant populations all underline the importance of developing an efficient and versatile transformation protocol for functional genomics studies. In barley, many types of explant tissues have been used for tissue culture and plant regeneration, and immature embryos have proven to be the most suitable for barley transformation. Immature embryos were first used as explants for barley transformation in 1986 [76], and gradually became the most popular system. However, plant regeneration from immature embryo-derived callus is influenced by genotype, with the highest rates of success having been obtained in the cv. Golden Promise [77]. Up to now, a variety of DNA delivery methods, which involve biological, chemical, mechanical and/or physical treatment, have proven effective in barley. However, *Agrobacterium*-mediated transformation seems to be the best strategy, since it is characterised by low cost, high efficiency, simple integration, stable inheritance and expression of the transgene over generations. Indeed, the current transformation protocol integrates *Agrobacterium* and immature embryos, yielding an average transformation efficiency of 25% [78]. This protocol is widely used for overexpression, RNAi (RNA interference) applications and, more recently, CRISPR/Cas 9-mediated gene editing, as discussed below. Transgenic approaches have been employed to control pathogens such as Barley Yellow Dwarf Virus (BYDV; [79]), *Fusarium graminearum* [80], leaf stripe disease (*Pyrenophora graminea*; [81]), powdery mildew (*Blumeria graminis* f. sp. *Hordei*; [82]) and stem rust (*Puccinia graminis* f. sp. *Tritici*; [83]). Moreover, the transgenic technology has also been used to increase tolerance to environmental stresses, such as drought [84,85] and frost [86,87,88], and to modify enzymes, such as α-amylase, β-amylase, β-glucanase and (1,3;1,4)-β-D-glucan endohydrolase [89,90,91,92,93], which have an influence on the brewing process. The transgenic technology is also essential for mutagenesis. In barley, insertional mutagenesis has been used to produce loss-of-function mutations based on transposable elements such as the Ac/Ds-based tagging system [94,95,96], and gain-of-function mutations using the activation tagging strategy that promotes or enhances, through random genomic insertion, the expression of neighbouring regions [95]. However, unlike the case in *Arabidopsis*, no large-scale T-DNA insertional populations are currently available for barley.

### 4.4. Induced Genetic Diversity: Gene Editing

The availability of the whole genome sequence, together with the recently developed gene-editing strategies, makes targeted mutagenesis possible in barley [97]. Among the various customized endonucleases used in plant research, the type II Clustered Regularly Interspaced Short Palindromic repeat (CRISPR)/CRISPR-associated protein 9 (Cas9) system has proven to be the best tool for gene editing [98]. The CRISPR/Cas9 gene-editing technology is easy to design and very precise. It requires the synthesis of oligonucleotides which, once transcribed into RNA, guide the Cas9 enzyme to the desired target. Being based on RNA–DNA interaction, the method is quite specific. The CRISPR/Cas9 technology can be used to create null alleles (i.e., gene knock-outs), and by adding a designed DNA template to the CRISPR/Cas9 system, it is possible to replace the target sequence via the error-free homology-directed repair pathway [99]. Furthermore, the system can be used to modulate gene expression [100]. In the past few years, the CRISPR/Cas9 technique has been increasingly applied to barley. In particular, a simple and efficient CRISPR/Cas9 platform for the induction of single and multiple, heritable mutations has been introduced [101]. The CRISPR/Cas9 technique has also been utilised to study genes involved in responses to pathogens, such as *HvMORC1*, whose protein product is one of the seven MORC members encoded by the barley genome [102]. Knock-out alleles of *HvCKX1* or *HvCKX3*, which are involved in the regulation of cytokinin metabolism and root morphology [103], as well as mutants in the *D-hordein* gene, which participates in the control of grain size and grain composition in barley [104], were also generated with the aid of CRISPR/Cas9. 

## 5. Barley is Ready for a New Age of Functional Genomics Studies and Genetic Improvements

**Figure 2:**
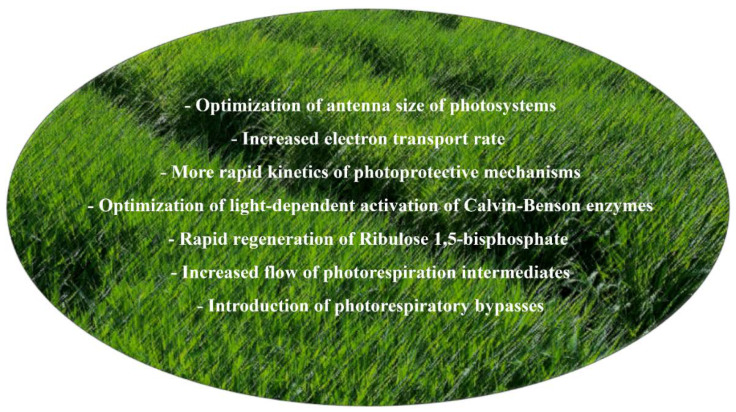
Biotechnological strategies that have been shown to enhance photosynthetic efficiency in model species. All of them can be applied to barley using the available genetic tools, and can potentially be improved by exploiting the genetic diversity of barley.

With the aid of the recently acquired collection of functional genomics tools, in a large part described above, the unique potential of barley as an ideal system for functional genomics studies can now be fully exploited. These new methods can elucidate, for instance, the molecular mechanisms behind chloroplast-to-nucleus communication, which is essential for chloroplast biogenesis and leaf emergence, leaf senescence and adaptation to environmental stresses. They can also be used to test—in an established crop plant—strategies intended to increase photosynthesis efficiency and biomass production, which have been shown to work in model species (Figure 2).

### 5.1. Plastid-to-Nucleus Retrograde Signalling

The chloroplast genome in barley encodes only 78 of the 3000 proteins that compose the plastid proteome [105,106]. The rest now reside in the nuclear DNA. Hence, signalling pathways that allow plastid and nuclear genomes to communicate with each other are essential for proper chloroplast development and functionality. The plastid-to-nucleus component of this circuit is often referred as “retrograde signalling” and it was first discovered in the barley mutant *albostrians* (see Section 1.2), which lacks plastid ribosomes and, concomitantly, shows reduced amounts and/or activities of nuclear-encoded plastidic proteins [33,34,35]. This channel is used to keep the nucleus informed of the developmental state of plastids (known as biogenic control), but it also signals changes in the functional status of fully developed chloroplasts in response to environmental factors, a process termed operational control [107]. Thus, chloroplast-to-nucleus communication is vital for chloroplast biogenesis and leaf emergence, as well as for the transition from chloroplast to gerontoplast during leaf senescence. Since the modulation of early leaf emergence and leaf senescence extends the proportion of the photosynthetically active radiation (PAR) that is intercepted by the crop over the growing season (the interception efficiency), both aspects of this communication are likely to be important determinants of crop yield. In addition, leaf senescence is a central process in maximising the efficiency of nutrient use, i.e., the ability of the plant to mobilise and translocate nutrients from leaves to grains. This is particularly true for small-grained cereals like barley, where up to 90% of the nitrogen is mobilised to the grains, mainly from the photosynthetic apparatus present in the leaves, and including Rubisco.

During the last 15 years, studies performed mainly in *Arabidopsis thaliana* have revealed a complex network of signals that allows chloroplasts to communicate their functional status to the nucleus. Singlet oxygen [108], H_2_O_2_ [109], the redox state of the photosynthetic electron transport chain [110], 3′-phosphoadenosine 5′-phosphate [111], the isoprenoid precursor methylerythritol cyclodiphosphate [112], β-cyclocitral [113,114] and other potential candidates [115,116,117,118,119] have been added to the list of operational control signals [107]. Understanding the degree to which these pathways are operative in monocot species like barley, together with a deeper knowledge of the regulatory, biochemical and redox networks that control the stability, functionality and disassembly of the photosynthetic apparatus under stress conditions and during induced senescence is pivotal for the identification of novel genes and favourable allelic variants for use in breeding programs. 

Chloroplast biogenesis during leaf emergence is initiated upon light perception and is also dependent on plastid retrograde signals. Over the past two decades, many publications have explored the role of plastid gene expression and tetrapyrrole biosynthesis as sources of biogenic signals. This system is disrupted in ‘genomes uncoupled’ mutants (*gun*; [120]). Five of the six GUN proteins (GUN2-6) are enzymes of the tetrapyrrole biosynthesis pathway and control the branched pathways downstream of Protoporphyrin IX (for a review see [121]). GUN1, however, does not take part in tetrapyrrole biosynthesis, but is required for the generation of retrograde signals triggered by the accumulation of tetrapyrrole precursors and inhibition of plastid gene expression (PGE) [122]. More recently, GUN1 has been reported to play a prominent role in the maintenance of chloroplast protein homeostasis by modulating plastid protein synthesis through its interaction with the plastid ribosomal protein S1 [123], and to control the activity of the plastid protein import machinery [124,125], suggesting that unimported preproteins in the cytosol could act as messenger molecules. Although most of the information on biogenic retrograde signalling has been obtained in Arabidopsis, the barley mutant *albostrians* has proved to be a valuable system for studying the regulation of tetrapyrrole biosynthesis and the involvement of these compounds in communication between plastids and the nucleus. Due to the lack of plastid-encoded proteins in this mutant, low levels of tRNA^Glu^, which serves as a substrate activator in tetrapyrrole biosynthesis, are observed in bleached *albostrians* leaves, and this might be one reason for the much lower chlorophyll content in *albostrians* plastids [126,127,128]. In the mutant, the common precursors of all tetrapyrroles are channelled in the direction of heme synthesis, while the formation of chlorophylls is repressed [127]. This, in turn, suggests that excess heme might leave the chloroplast and act as a signalling molecule, as has been observed in Arabidopsis [129]. Recently, the mutation responsible for the *albostrians* phenotype has been identified. It lies in the barley gene *HvCMF7*, which codes for a putative plastid protein that belongs to the CCT motif family (CMF), which includes CONSTANS, CO-like and TOC1 [130]. This gene is likely to play a crucial role in plastid ribosome formation during early embryo development and hence for chloroplast development. The identification of the gene defect that causes the *albostrians* phenotype represents a major step forward in the understanding of the molecular mechanisms that mediate chloroplast biogenesis in barley. In this context, it would be interesting to determine whether a GUN1-like protein exists in barley. Furthermore, the gradient in chloroplast biogenesis observed in the barley leaf blade provides access to leaf sectors that contain cells of the same developmental stage, which facilitates the use of RNA-seq and proteomics approaches to investigate the molecular network at the basis of proplastid-to-chloroplast differentiation.

### 5.2. Photosynthesis and Yield

Doubling agricultural production by 2050 is essential if the demands of a constantly growing population for food and biomass are to be satisfied. Among cereals, barley straw is characterised by the highest content of carbohydrates [131]. Barley is therefore an ideal feedstock for the bio-based economy, since it can be used for the production of food/feed/spirits from grains, and renewable resources, including biofuel, from straw. It is worth mentioning here that none of the huge improvements in agricultural production made during the ‘Green Revolution’ were directly related to manipulations of photosynthesis. Hence, the process remains an unexplored target with a high potential for crop improvement. Indeed, the theoretical maximum efficiency of the conversion of solar energy into biomass in a C3 crop like barley is around 4.6% and this value decreases to 2.4% under field conditions across the entire growing season. Therefore, the conversion efficiency of visible solar energy is considerably below its theoretical maximum, and several promising targets for its improvement have been identified in model species, some of which are described below (Figure 2 and Table 4).

### 5.3. Optimization of Antenna Size in Crop Canopies

One of the main reasons for the lower conversion efficiency of solar radiation is the saturation of the photosynthetic machinery with light. Indeed, it has been demonstrated that the photosynthetic apparatus operates with near maximum efficiency when light levels are low. For instance, the photosynthetic process in a C3 plant is already saturated at approximately 25% of maximum sunlight [153]. As light absorption increases, photosynthetic efficiency declines. In fact, the antennal apparatus of photosystems is larger than optimal, since under competitive natural conditions, shading of neighbouring plants confers an important selective advantage on the upper storey [154]. However, this behaviour is clearly disadvantageous for cultivated crops. Reducing the size of the antenna systems in the leaves of the upper canopy can offer important advantages, saving the metabolic resources required for the production of the antenna complex and the activity of photoprotective mechanisms, while increasing the amount and quality of light able to reach the lower leaves [155]. Several studies have provided evidence that the reduction of antenna size can improve photosynthetic efficiency. For instance, cell suspensions of *Synechocystis* PCC6714 and *Chlorella pyrenoidosa* with reduced contents of light-harvesting pigments showed a photosynthetic activity 20–30% higher than the wild type [156]. An engineered *Chlamydomonas reinhardtii* strain with a small PSII antenna size exhibited about a 50% increase in photosynthetic efficiency under saturating levels of light [157]. In the same alga, a partial reduction in chlorophyll b levels resulted in a two-fold increase in photosynthetic rate at high light intensities [158]. The hypothesis that a constitutively smaller antenna size should improve canopy photosynthetic efficiency by minimizing the over-absorption of the incident sunlight, and improving canopy light penetration, has also been tested in higher plants. A decrease in antenna size in tobacco, for instance, led to an increase of about 25% in plant–canopy biomass accumulation under high-density cultivation conditions [39]. Similarly, beneficial effects were observed in a rice genotype with pale green leaves cultivated under high light conditions [132].

### 5.4. Increased Photosynthetic Electron Transport 

The modification of the thylakoid electron transport chain has also been reported to contribute to the improvement of photosynthetic performance and biomass accumulation. For instance, Chida et al. [133] showed that the expression of the algal *Porphyra yezoensis* cytochrome *c_6_* in the chloroplasts of Arabidopsis led to an increase of CO_2_ assimilation, and biomass production [133]. The overexpression of cytochrome *c_6_* from *Ulva fasciata* in tobacco gave similar results [134]. Moreover, overexpression of plastocyanin in Arabidopsis resulted in 1.6-fold increase in leaf area [135]. A large increase in biomass and seed yield was also obtained in Arabidopsis upon overexpression of its endogenous Rieske FeS protein, a subunit of the cytochrome *b_6_/f* [136].

### 5.5. Improving the Adaptation to Fluctuating Light: Dissipation of Excess Energy through Non-Photochemical Quenching

Non-Photochemical Quenching (NPQ) serves as photoprotective mechanism in leaves, and is responsible for the dissipation of excess absorbed light energy as heat, thus preventing oxidative damage. Dissipation takes place in photosystem II (PSII) and involves the enzymes violaxanthin de-epoxidase (VDE) and zeaxanthin epoxidase (ZEP) [159], together with PsbS, a PSII protein subunit [160]. Activation and relaxation of NPQ take place over timescales of seconds to minutes, which are rather slow with respect to the instantaneous changes in light intensities observable within plant canopies in field settings. This leads to loss of photosynthetic efficiency, as heat dissipation continues even when light does not exceed the photosynthetic capacity [161]. Recently, the overexpression of *PsbS*, *ZEP* and *VDE* genes was reported in tobacco plants. These plants displayed an improved kinetics of NPQ, resulting in about 20% increase in biomass accumulation under both greenhouse and field conditions [137].

### 5.6. Transgenic Manipulation of the Calvin–Benson Cycle

Attempts to improve photosynthetic efficiency through transgenic manipulations have also focused on the overexpression of single enzymes of the Calvin–Benson cycle (Table 4). For example, overexpression of sedoheptulose-1,7-bisphosphatase (SBPase) in *Arabidopsis* [139], tobacco [138,140] and tomato [141] has shown that an increased SBPase activity results in a 30-40% increase in biomass yield, depending on the species. More recently it was shown that significant increases in photosynthetic rates, biomass and grain yield can be achieved by augmenting SBPase activity in wheat [162]. In 2012, the overexpression of the fructose 1,6-bisphosphate aldolase (FBPA) enzyme in tobacco also resulted in an increase in biomass production of 10-30% [142]. Overall, these findings demonstrated that SBPase and FBPA are enzymes that can exert control over the flow of carbon in the Calvin–Benson cycle in a number of different species, and proved that their manipulation also benefits grain yield. Efforts to increase the light activation rate of the Calvin–Benson cycle have also yielded very promising results. For instance, the overexpression of maize Rubisco activase in rice increased the rate of Rubisco activation by light and at high temperature (40 °C; [143]). Increased levels of thioredoxin *f* (TRX *f*), which is known to reductively activate enzymes of the Calvin–Benson cycle, have also increased leaf weight and sugar content under both ambient and increased CO_2_ conditions [144,145]. Overexpression of the chloroplast NADPH-dependent thioredoxin reductase (NTRC), also reported to interact with several Calvin–Benson enzymes, has also been shown to be beneficial for productivity in Arabidopsis. Indeed, the biomass increases in the NTRC-overexpressing Arabidopsis plants were between 2- and 2.5-fold in plants grown under long- and short-day conditions, respectively, at fluence levels of 600 μmol m^−2^ s^−1^ light [146,147]. Additionally, overexpression of NTRC has been reported to enhance tolerance to oxidative and drought stresses. These are traits of great significance for improvement of crop productivity under field conditions [163].

### 5.7. Photorespiration and Photorespiratory Bypasses

Photorespiration is also an important target to improve photosynthesis. For instance, the reversible conversion of glycine into serine that takes place in mitochondria is crucial for plants [164,165,166,167,168,169]. These reactions involve the pyridoxal phosphate-dependent enzyme glycine decarboxylase (P-protein), the THF-dependent enzyme aminomethyltransferase (T-protein), the NAD^+^-dependent enzyme dihydrolipoyl dehydrogenase (L-protein) and the lipoic acid-containing H-protein. In Arabidopsis, overexpression of the H- or L-protein resulted in an improvement in photosynthetic efficiency and larger biomass accumulation [148,149]. Similar results were also obtained with the mesophyll-specific overexpression of the H-protein in tobacco [150]. Besides increasing photorespiration flow and reducing accumulation of photorespiratory intermediates, the most promising strategies for enhancing productivity are based on photorespiratory bypasses, i.e., the introduction of alternative pathways to metabolize 2PG, thus liberating CO_2_ in the chloroplast stroma for Rubisco fixation [151,152]. In particular, Kebeish et al. introduced the *Escherichia coli* glycolate catabolic pathway into Arabidopsis. In these transgenic plants, the glycolate derived from the dephosphorylation of 2-phosphoglycolate was converted into glycerate in the chloroplast without the release of ammonia, which can make nitrogen use more efficient. Moreover, CO_2_ release was shifted from mitochondria to chloroplasts, based on the idea that CO_2_ should have a better chance to be re-fixed by Rubisco if it is released in the chloroplast rather than in the mitochondria. As a result of the increased concentration of CO_2_ in the chloroplasts and the reduced energy demand for photorespiration, transgenic plants grew faster and produced more biomass, indicating that the bypass effectively reduced photorespiration and enhanced photosynthesis. Inspired by this work, another group [152] introduced synthetic glycolate metabolic pathways that are more efficient than the endogenous pathway into tobacco chloroplasts. Flux through the synthetic pathways was maximized by inhibiting glycolate export from the chloroplast. These synthetic pathways were able to improve photosynthetic quantum yield by 20% and biomass productivity by >40% in replicated field trials. 

It is reasonable to expect that the various transgenic approaches described above will result in increased photosynthetic quantum yield, biomass and, eventually, grain yield also in barley, although species-dependent effects were observed in the multigene manipulation of the Calvin–Benson cycle [138,139]. In addition to that, the large genetic diversity readily available in barley also allows the exploitation of natural and/or induced allelic variants of enzymes involved in defining the antenna size of photosystems, in thylakoid electron transport, NPQ, the Calvin–Benson cycle, and photorespiration, which could ameliorate barley yield (Table 4). These allelic variants can be identified either by allele mining of exome sequences of barley cultivars, landraces and wild varieties (see Table 2), or through TILLING of mutant populations (Table 3). As mentioned above, the latter approaches enable one to obtain new barley varieties by using the classical breeding approach based on crosses. Thus, the performance of the new varieties can be verified under field conditions and they could be grown even in countries that have banned the cultivation of genetically modified plants.

## 6. Conclusions

Its genetic diversity and the availability of a large collection of molecular tools make barley an ideal model crop for functional genomics studies related to chloroplast biogenesis and retrograde communication. Such studies will reveal to what extent retrograde signalling mechanisms are conserved between Arabidopsis and barley, and permit us to learn more about aspects of chloroplast biogenesis that are specific to monocots. The recent identification of the genetic factor responsible for the *albostrians* phenotype demonstrates that this type of analysis can now be effectively conducted in barley. The fact that the gene concerned, *HvCMF7*, encodes a protein that is apparently located exclusively in plastids highlights the need for systematic investigation of barley mutants with defects in chloroplast biogenesis. Furthermore, novel approaches to the screening of barley mutant populations are required to elucidate the molecular details of the chloroplast-to-nucleus communication. The genes and allelic variants identified in future studies could have an important impact in breeding programs, since retrograde communication controls the leaf life cycle. 

Barley can also make a significant contribution to the testing of novel biotechnological strategies for improving photosynthesis, and the validation of their effects on biomass accumulation and grain yield. In recent decades, our knowledge of the photosynthetic process has increased substantially, and improvements in its efficiency have been demonstrated in different model species. The high level of conservation of the photosynthetic process strongly argues that similar enhancements can be achieved in barley. Thanks to the high content of sugars in the straw, barley could be transformed into a dual-purpose crop suitable for the production of biofuel from the straw, and food, feed and spirits from the grain. Furthermore, the use of barley varieties characterised by high photosynthetic efficiency and reduced antenna size of photosystems is a promising strategy for boosting productivity and water use efficiency, while increasing land–surface reflectivity to offset greenhouse gas warming. In light of the foreseeable rise in the demand for food by the middle of this century, and the fact that the development and commercialization of a new plant variety with improved quality takes 10 to 15 years, concerted efforts to increase agricultural yields through manipulation of photosynthesis must be initiated immediately. The “redesign” of photosynthesis must represent one of the main pillars of the next “Green Revolution”.

## Figures and Tables

**Table 1:** List of databases, genome browsers and bioinformatics tools available for barley genome analyses.

Tool	Description/Application	URL	Reference
**BARLEX**	The Barley Genome Explorer permits visual inspection of BAC overlaps, and comparisons of BACs and provides useful information on genes and markers	http://barlex.barleysequence.org	[49]
**EnsemblPlants**	A genome browser that incorporates genomic data from diverse organisms, including numerous plant species. It enables users to compare genome-scale datasets with the aid of a single collection of interfaces	http://plants.ensembl.org	[50]
**IPK Barley BLAST Server**	Barley BLAST server for genome-scale homology-based searches	http://webblast.ipk-gatersleben.de/barley	[51]
**Golden Promise Genome**	GMAP and BLAST server for barley (cv. Golden Promise) genome comparisons, including mapping of transcripts	https://ics.hutton.ac.uk/gmapper/	[48]
**Gramene**	Integrated data resource for comparative functional genomics in crops and model plant species	http://www.gramene.org	[52]
**PlantsDB**	Provides data and information resources for individual plant species and a platform for integrative and comparative plant genome research.	http://pgsb.helmholtz-muenchen.de/plant/	[53]
**BaRTv1.0**	Barley Reference Transcript Dataset provides access to 177,240 barley-expressed transcripts covering 60,444 genes	https://ics.hutton.ac.uk/barleyrtd/	[54]

**Table 2:** List of representative collections of natural variants of barley available at different institutions worldwide, together with online platforms that provide information about barley genetic resources.

**Collections of natural genetic diversity**		
**Gene Bank**	**Country**	**URL**
**PGRC** Plant Gene Resources of Canada, Saskatoon Research Centre, Agriculture and Agri-Food Canada)	Canada	https://pgrc.agr.gc.ca/index_e.html
**NSGC** The National Small Grains Collection is part of the National Plant Germplasm System (NPGS) of the United States Department of Agriculture - Agricultural Research Service (USDA-ARS)	USA	https://www.ars.usda.gov/pacific-west-area/aberdeen-id/small-grains-and-potato-germplasm-research/docs/barley-wheat-genetic-stocks-collections-1/
**ICARDA** International Centre for Agricultural Research in the Dry Areas	Global	https://grs.icarda.org/
**IPK** Leibniz Institute of Plant Genetics and Crop Plant Research	Germany	http://gbis.ipk-gatersleben.de
**WHEALBI** WHEAt and barley Legacy for Breeding Improvement	France	http://wheat-urgi.versailles.inra.fr/Projects/Achieved-projects/Whealbi
**NORDGEN** Nordic Genetic Resources Centre	Sweden	https://www.nordgen.org/bgs/
**GRU** Germplasm Resource Unit, John Innes Centre	UK	https://www.seedstor.ac.uk/
**NARO** NIAS, National Institute of Agrobiological Sciences	Japan	https://www.gene.affrc.go.jp/databases_en.php
**Online platforms for barley germplasm searches**		
**Name**	**Description**	**URL**
**GENESIS**	An online platform containing information about plant genetic resources for food and agriculture, conserved in gene banks worldwide	https://www.genesys-pgr.org/
**SINGER (The system-wide Information Network for Genetic Resources)**	An online catalogue of crop collections together with their locations	https://www.gbif.org/dataset/85818aea-f762-11e1-a439-00145eb45e9a
**EURISCO (The European Search Catalogue for Plant Genetic Resources)**	Information on more than 2 million crop plant accessions and their wild relatives, preserved ex situ by almost 400 institutes in Europe and beyond	https://www.ecpgr.cgiar.org/resources/germplasm-databases/eurisco-catalogue/

**Table 3:** List of representative barley mutant populations obtained by either chemical or physical mutagenesis in different genetic backgrounds, i.e., cultivars.

Induced Mutant Populations		
Cultivar	Mutagen	Reference
Optic	EMS	[60]
Barke	EMS	[64]
Morex	NaN_3_	[61]
Lux	NaN_3_	[65]
DH-930-36	MNU	[66]
DH-930-36	Gamma rays	[66]
Sebastian	NaN_3_+MNU	[62]
Golden Promise	EMS	[63]

**Table 4:** Brief summary of biotechnological strategies that are being employed for photosynthesis improvement in barley.

Target	Efficiency Gain	Strategy	Outcome	References
Retrograde signalling				
1. Investigating the existence of a GUN1-dependent retrograde signalling pathway in barley	Not expected	Knock-out of the HORVU.MOREX.r2.5HG0366860.1 gene through gene editing	Molecular details of the retrograde signalling pathway involved in chloroplast biogenesis with possible repercussions for the control of leaf life-cycle	[122]
Light phase of photosynthesis				
1. Optimisation of the antenna size	20–50%	Reduction of photosystem antenna size obtained by either reducing chlorophyll production or decreasing levels of the photosystem antenna proteins by gene editing or introgression of induced mutations. Identification of allelic variants by allele mining and TILLING.	More uniform photosynthetic performance throughout the crop canopy and prevention of photo-oxidative damage in the upper layers of the canopy. Increases in land–surface reflectivity to offset greenhouse gas warming.	[39,132]
2. Increased photosynthetic electron transport	30–70%	Increased accumulation of electron carriers, such as cytochrome *c_6_*, plastocyanin and Rieske proteins, by transgenic approaches. Identification of allelic variants by allele mining and TILLING.	Increased electron transport rate through the thylakoid membranes	[133,134,135,136]
3. Fine-Tuning of NPQ	30%	Increased accumulation of VDE, ZEP and PsbS by transgenic approaches. Identification of allelic variants by allele mining and TILLING.	More rapid induction and relaxation of heat dissipation at PSII.	[137]
Dark phase of photosynthesis				
1. Increasing the abundance of different enzymes of the Calvin–Benson cycle	>30%	Increased accumulation of SBPase and FBPA enzymes by transgenic approaches. Identification of allelic variants by allele mining and TILLING.	Optimization of ribulose 1,5-bisphosphate (RuBP) regeneration.	[138,139,140,141,142]
2. Increasing the efficiency of light activation of Calvin–Benson enzymes	>20%	Increased accumulation of Rubisco activase, TRX *f* and NTRC by transgenic approaches. Identification of allelic variants by allele mining and TILLING.	More efficient light-dependent activation of Calvin–Benson enzyme optimises CO_2_ fixation.	[143,144,145,146,147]
Photorespiration				
1. Increasing the photorespiration flow of intermediates	>15%	Increased accumulation of H- and L-proteins by transgenic approaches.	Reduced accumulation of photorespiration intermediates and increased CO_2_ assimilation rate	[148,149,150]
2. Synthetic bypasses to photorespiration	>20%	Introduction of natural and synthetic glycolate catabolic pathways in the chloroplast	Increased CO_2_ assimilation rates	[151,152]
